# (*E*,*E*)-3,3′-Dimethyl-1,1′-diphenyl-4,4′-{[3-aza­pentane-1,5-diylbis(aza­nedi­yl)]bis­(phenyl­methyl­idyne)}di-1*H*-pyrazol-5(4*H*)-one

**DOI:** 10.1107/S1600536810048981

**Published:** 2010-11-27

**Authors:** Zhao-Po Zhang, Yuan Wang, Xiao-Xia Li, Yan-Wei Li

**Affiliations:** a Department of Physics and Chemistry, Henan Polytechnic University, Jiaozuo 454000, People’s Republic of China; b Institute of Functional Materials, Jiangxi University of Finance & Economics, Nanchang 330013, People’s Republic of China

## Abstract

The asymmetric unit of the title compound, C_38_H_37_N_7_O_2_, contains one half-mol­ecule, situated on a twofold rotational axis, in which one amino group is involved in intra­molecular N—H⋯O hydrogen bond and the two phenyl rings are twisted from the plane of pyrazolone ring by 26.69 (10) and 79.64 (8)°. The crystal packing exhibits no classical inter­molecular contacts.

## Related literature

For the synthesis of the title compound and the DNA binding properties of its transition metal complexes, see: Yang *et al.* (2000 ▶); Wang & Yang (2005 ▶). For the similar structure of (*E*,*E*)-3,3′-dimethyl-1,1′-diphenyl-4,4′-{(ethane-1,2-diyldiimino)­bis­[(2-fur­yl)-methyl­idyne]}di-1*H*-pyrazol-5(4*H*)-one, see: Wang (2010 ▶).
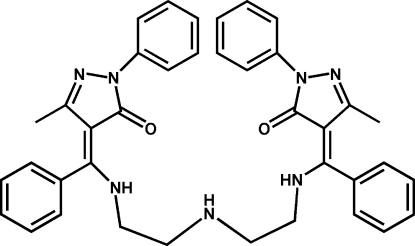


## Experimental

### 

#### Crystal data

C_38_H_37_N_7_O_2_
*M* *_r_* = 623.75
Monoclinic, 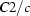

*a* = 20.3219 (8) Å
*b* = 8.1990 (2) Å
*c* = 20.5468 (6) Å
β = 106.748 (2)°
*V* = 3278.27 (18) Å^3^
*Z* = 4
Mo *K*α radiation
μ = 0.08 mm^−1^
*T* = 296 K
0.23 × 0.21 × 0.15 mm

#### Data collection

Bruker SMART APEXII CCD diffractometer
Absorption correction: multi-scan (*SADABS*; Bruker, 2007 ▶) *T*_min_ = 0.982, *T*_max_ = 0.988
8269 measured reflections
3765 independent reflections
1886 reflections with *I* > 2σ(*I*)
*R*_int_ = 0.021

#### Refinement

*R*[*F* ^2^ > 2σ(*F* ^2^)] = 0.054
*wR*(*F* ^2^) = 0.179
*S* = 1.01
3765 reflections
214 parameters
H-atom parameters constrained
Δρ_max_ = 0.34 e Å^−3^
Δρ_min_ = −0.29 e Å^−3^

### 

Data collection: *APEX2* (Bruker, 2007 ▶); cell refinement: *SAINT* (Bruker, 2007 ▶); data reduction: *SAINT*; program(s) used to solve structure: *SHELXS97* (Sheldrick, 2008 ▶); program(s) used to refine structure: *SHELXL97* (Sheldrick, 2008 ▶); molecular graphics: *SHELXTL* (Sheldrick, 2008 ▶); software used to prepare material for publication: *SHELXTL*.

## Supplementary Material

Crystal structure: contains datablocks I, global. DOI: 10.1107/S1600536810048981/cv5005sup1.cif

Structure factors: contains datablocks I. DOI: 10.1107/S1600536810048981/cv5005Isup2.hkl

Additional supplementary materials:  crystallographic information; 3D view; checkCIF report

## Figures and Tables

**Table 1:** Hydrogen-bond geometry (Å, °)

*D*—H⋯*A*	*D*—H	H⋯*A*	*D*⋯*A*	*D*—H⋯*A*
N3—H3*A*⋯O1	0.86	2.00	2.722 (2)	140

**Fig. 1.**
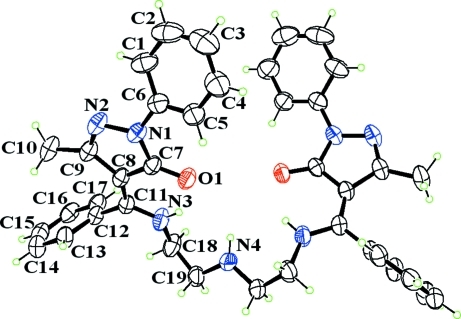
The molecular structure shown with 30% probability displacement ellipsoids. Unlabelled atoms are related with the labelled ones by symmetry operation (-x, y, -z+1/2).

## supplementary crystallographic information

### Comment

The Schiff bases derivatives of 1-phenyl-3-methyl-4-benzoyl-5-pyrazolone (PMBP) and their metal complexes have been widely studied because of their high biological and pharmaceutical activities (Yang *et al.,* 2000; Wang *et al.,* 2005). For our interest in coordination chemistry, the crystal structure of the title compound was determined by X-ray diffraction analysis.

The asymmetric unit of the title compound contains a half of the molecule situated on a twofold rotational axis. In the independent part of the molecule, one amino group is invloved in intramolecular N—H···O hydrogen bond (Table 1), and two phenyl rings are twisted from the plane of pyrazolone ring at 26.69 (10)° and 79.64 (8)°, respectively. The crystal packing exhibits no classical intermolecular contacts.

### Experimental

The title compound was prepared according to the literature (Wang *et al.*, 2005). 1.1 g (4 mmol) of PMBP were dissolved in EtOH (50 ml), and the EtOH solution containing diethylenetriamine (0.2 g, 2 mmol) was added dropwise. The mixture refluxed on a water bath for 4 h, then part of the solvent was removed on a rotary evaporator. After cooling to 273 K, a large amount of yellow precipitate separated out. Yellow block crystals were obtained by slow evaporation of an ethanol /chloroform (1:1) solution.

### Refinement

All H atoms were placed in calculated positions, with C—H = 0.93–0.97 Å and N—H = 0.86 Å, and were treated as riding, with *U*_iso_(H) = 1.2-1.5 U_eq_ of the parent atom.

### Crystal data

**Table:**

C_38_H_37_N_7_O_2_	*F*(000) = 1320
*M**_r_* = 623.75	*D*_x_ = 1.264 Mg m^−^^3^
Monoclinic, *C*2/*c*	Mo *K*α radiation, λ = 0.71073 Å
*a* = 20.3219 (8) Å	Cell parameters from 1721 reflections
*b* = 8.1990 (2) Å	θ = 2.5–22.0°
*c* = 20.5468 (6) Å	µ = 0.08 mm^−^^1^
β = 106.748 (2)°	*T* = 296 K
*V* = 3278.27 (18) Å^3^	Block, yellow
*Z* = 4	0.23 × 0.21 × 0.15 mm

### Data collection

**Table:**

Bruker SMART APEXII CCD diffractometer	3765 independent reflections
Radiation source: fine-focus sealed tube	1886 reflections with *I* > 2σ(*I*)
graphite	*R*_int_ = 0.021
phi and ω scans	θ_max_ = 27.7°, θ_min_ = 2.1°
Absorption correction: multi-scan (*SADABS*; Bruker, 2007)	*h* = −26→20
*T*_min_ = 0.982, *T*_max_ = 0.988	*k* = −8→10
8269 measured reflections	*l* = −26→25

### Refinement

**Table:**

Refinement on *F*^2^	Primary atom site location: structure-invariant direct methods
Least-squares matrix: full	Secondary atom site location: difference Fourier map
*R*[*F*^2^ > 2σ(*F*^2^)] = 0.054	Hydrogen site location: inferred from neighbouring sites
*wR*(*F*^2^) = 0.179	H-atom parameters constrained
*S* = 1.01	*w* = 1/[σ^2^(*F*_o_^2^) + (0.0884*P*)^2^ + 0.1149*P*] where *P* = (*F*_o_^2^ + 2*F*_c_^2^)/3
3765 reflections	(Δ/σ)_max_ < 0.001
214 parameters	Δρ_max_ = 0.34 e Å^−^^3^
0 restraints	Δρ_min_ = −0.29 e Å^−^^3^

### Special details

**Table:**

Geometry. All e.s.d.'s (except the e.s.d. in the dihedral angle between two l.s. planes) are estimated using the full covariance matrix. The cell e.s.d.'s are taken into account individually in the estimation of e.s.d.'s in distances, angles and torsion angles; correlations between e.s.d.'s in cell parameters are only used when they are defined by crystal symmetry. An approximate (isotropic) treatment of cell e.s.d.'s is used for estimating e.s.d.'s involving l.s. planes.
Refinement. Refinement of *F*^2^ against ALL reflections. The weighted *R*-factor *wR* and goodness of fit *S* are based on *F*^2^, conventional *R*-factors *R* are based on *F*, with *F* set to zero for negative *F*^2^. The threshold expression of *F*^2^ > σ(*F*^2^) is used only for calculating *R*-factors(gt) *etc*. and is not relevant to the choice of reflections for refinement. *R*-factors based on *F*^2^ are statistically about twice as large as those based on *F*, and *R*- factors based on ALL data will be even larger.

### Fractional atomic coordinates and isotropic or equivalent isotropic displacement parameters (Å^2^)

**Table:**

	*x*	*y*	*z*	*U*_iso_*/*U*_eq_	
O1	0.06573 (8)	0.23883 (18)	0.35444 (8)	0.0809 (5)	
N1	0.14838 (9)	0.0606 (2)	0.41990 (8)	0.0707 (5)	
C7	0.12657 (11)	0.1980 (3)	0.38051 (10)	0.0645 (6)	
C12	0.25215 (10)	0.4844 (3)	0.32781 (11)	0.0661 (6)	
N2	0.21985 (10)	0.0439 (3)	0.43823 (10)	0.0819 (6)	
N3	0.12874 (9)	0.4629 (2)	0.29494 (10)	0.0833 (6)	
H3A	0.0927	0.4145	0.2992	0.100*	
C11	0.18821 (10)	0.4066 (3)	0.33364 (11)	0.0657 (6)	
N4	0.0000	0.6106 (3)	0.2500	0.0898 (9)	
H4	0.0000	0.5057	0.2500	0.108*	
C8	0.18835 (10)	0.2749 (3)	0.37639 (10)	0.0635 (6)	
C6	0.10856 (13)	−0.0732 (3)	0.42954 (10)	0.0719 (6)	
C17	0.27276 (12)	0.4657 (3)	0.27000 (11)	0.0738 (6)	
H17	0.2456	0.4070	0.2333	0.089*	
C9	0.24330 (11)	0.1713 (3)	0.41344 (10)	0.0715 (6)	
C19	0.06028 (11)	0.7026 (3)	0.25289 (12)	0.0802 (7)	
H19A	0.0749	0.7608	0.2958	0.096*	
H19B	0.0500	0.7825	0.2165	0.096*	
C15	0.37336 (13)	0.6229 (3)	0.32006 (17)	0.0930 (8)	
H15	0.4144	0.6685	0.3174	0.112*	
C13	0.29269 (12)	0.5747 (3)	0.38196 (12)	0.0822 (7)	
H13	0.2791	0.5876	0.4212	0.099*	
C16	0.33348 (14)	0.5339 (3)	0.26650 (14)	0.0840 (7)	
H16	0.3476	0.5196	0.2277	0.101*	
C5	0.04144 (14)	−0.0517 (3)	0.42877 (12)	0.0830 (7)	
H5	0.0224	0.0524	0.4239	0.100*	
C18	0.11649 (12)	0.5955 (3)	0.24635 (13)	0.0913 (8)	
H18A	0.1048	0.5511	0.2007	0.110*	
H18B	0.1582	0.6592	0.2535	0.110*	
C14	0.35261 (13)	0.6447 (3)	0.37766 (15)	0.0951 (8)	
H14	0.3792	0.7068	0.4136	0.114*	
C10	0.31871 (12)	0.1867 (4)	0.42487 (13)	0.0961 (8)	
H10A	0.3412	0.0905	0.4472	0.144*	
H10B	0.3354	0.2803	0.4528	0.144*	
H10C	0.3281	0.1991	0.3820	0.144*	
C1	0.13730 (17)	−0.2275 (3)	0.43868 (12)	0.0930 (8)	
H1	0.1832	−0.2428	0.4409	0.112*	
C2	0.0968 (2)	−0.3578 (4)	0.44439 (14)	0.1196 (12)	
H2	0.1156	−0.4621	0.4496	0.144*	
C4	0.00213 (17)	−0.1842 (4)	0.43516 (14)	0.1080 (9)	
H4A	−0.0435	−0.1692	0.4344	0.130*	
C3	0.0297 (3)	−0.3380 (5)	0.44262 (15)	0.1220 (12)	
H3	0.0030	−0.4276	0.4464	0.146*	

### Atomic displacement parameters (Å^2^)

**Table:**

	*U*^11^	*U*^22^	*U*^33^	*U*^12^	*U*^13^	*U*^23^
O1	0.0582 (10)	0.0849 (11)	0.0935 (11)	0.0105 (8)	0.0123 (8)	0.0203 (9)
N1	0.0691 (12)	0.0775 (12)	0.0639 (11)	0.0186 (10)	0.0165 (9)	0.0105 (10)
C7	0.0633 (14)	0.0714 (13)	0.0550 (11)	0.0118 (11)	0.0113 (10)	0.0031 (11)
C12	0.0527 (12)	0.0789 (14)	0.0630 (13)	0.0104 (10)	0.0106 (10)	0.0064 (11)
N2	0.0710 (13)	0.1030 (15)	0.0698 (12)	0.0299 (11)	0.0171 (9)	0.0151 (11)
N3	0.0534 (11)	0.0994 (14)	0.0904 (14)	0.0020 (10)	0.0098 (9)	0.0312 (12)
C11	0.0534 (12)	0.0782 (14)	0.0613 (12)	0.0071 (11)	0.0099 (9)	−0.0004 (11)
N4	0.0586 (17)	0.0646 (15)	0.141 (3)	0.000	0.0209 (16)	0.000
C8	0.0578 (13)	0.0768 (14)	0.0529 (11)	0.0114 (11)	0.0109 (9)	0.0040 (11)
C6	0.0888 (17)	0.0736 (15)	0.0502 (11)	0.0087 (13)	0.0153 (11)	0.0050 (11)
C17	0.0676 (15)	0.0905 (16)	0.0623 (13)	0.0129 (12)	0.0171 (11)	0.0103 (12)
C9	0.0635 (14)	0.0937 (16)	0.0556 (12)	0.0222 (13)	0.0144 (10)	0.0047 (12)
C19	0.0640 (15)	0.0849 (15)	0.0790 (15)	−0.0046 (13)	0.0001 (11)	0.0105 (13)
C15	0.0587 (15)	0.0976 (19)	0.118 (2)	0.0060 (14)	0.0185 (15)	0.0314 (18)
C13	0.0626 (14)	0.1095 (18)	0.0691 (14)	0.0038 (14)	0.0101 (11)	−0.0065 (14)
C16	0.0793 (18)	0.0950 (17)	0.0848 (17)	0.0241 (14)	0.0348 (14)	0.0294 (15)
C5	0.0876 (18)	0.0819 (16)	0.0771 (15)	−0.0009 (14)	0.0201 (13)	0.0111 (13)
C18	0.0667 (15)	0.1080 (18)	0.0938 (17)	0.0109 (14)	0.0146 (12)	0.0381 (15)
C14	0.0646 (17)	0.110 (2)	0.101 (2)	−0.0025 (14)	0.0075 (14)	−0.0023 (16)
C10	0.0650 (16)	0.134 (2)	0.0863 (16)	0.0318 (15)	0.0163 (12)	0.0204 (16)
C1	0.136 (2)	0.0787 (17)	0.0666 (14)	0.0274 (17)	0.0325 (15)	0.0120 (13)
C2	0.205 (4)	0.074 (2)	0.0774 (19)	0.014 (2)	0.037 (2)	0.0158 (15)
C4	0.119 (2)	0.105 (2)	0.0920 (19)	−0.020 (2)	0.0188 (16)	0.0229 (17)
C3	0.182 (4)	0.100 (3)	0.0778 (18)	−0.037 (3)	0.026 (2)	0.0155 (17)

### Geometric parameters (Å, °)

**Table:**

O1—C7	1.243 (2)	C19—H19A	0.9700
N1—C7	1.383 (3)	C19—H19B	0.9700
N1—N2	1.398 (2)	C15—C16	1.374 (4)
N1—C6	1.411 (3)	C15—C14	1.378 (4)
C7—C8	1.429 (3)	C15—H15	0.9300
C12—C17	1.377 (3)	C13—C14	1.372 (3)
C12—C13	1.391 (3)	C13—H13	0.9300
C12—C11	1.483 (3)	C16—H16	0.9300
N2—C9	1.310 (3)	C5—C4	1.377 (4)
N3—C11	1.324 (3)	C5—H5	0.9300
N3—C18	1.448 (3)	C18—H18A	0.9700
N3—H3A	0.8600	C18—H18B	0.9700
C11—C8	1.392 (3)	C14—H14	0.9300
N4—C19	1.425 (3)	C10—H10A	0.9600
N4—C19^i^	1.425 (3)	C10—H10B	0.9600
N4—H4	0.8600	C10—H10C	0.9600
C8—C9	1.435 (3)	C1—C2	1.375 (4)
C6—C5	1.371 (3)	C1—H1	0.9300
C6—C1	1.384 (3)	C2—C3	1.362 (5)
C17—C16	1.375 (3)	C2—H2	0.9300
C17—H17	0.9300	C4—C3	1.370 (5)
C9—C10	1.487 (3)	C4—H4A	0.9300
C19—C18	1.477 (3)	C3—H3	0.9300
			
C7—N1—N2	111.73 (18)	C16—C15—H15	120.0
C7—N1—C6	127.98 (19)	C14—C15—H15	120.0
N2—N1—C6	118.64 (18)	C14—C13—C12	120.2 (2)
O1—C7—N1	125.6 (2)	C14—C13—H13	119.9
O1—C7—C8	129.6 (2)	C12—C13—H13	119.9
N1—C7—C8	104.77 (18)	C15—C16—C17	120.3 (2)
C17—C12—C13	119.5 (2)	C15—C16—H16	119.8
C17—C12—C11	120.9 (2)	C17—C16—H16	119.8
C13—C12—C11	119.66 (19)	C6—C5—C4	120.0 (3)
C9—N2—N1	106.44 (17)	C6—C5—H5	120.0
C11—N3—C18	128.4 (2)	C4—C5—H5	120.0
C11—N3—H3A	115.8	N3—C18—C19	111.5 (2)
C18—N3—H3A	115.8	N3—C18—H18A	109.3
N3—C11—C8	119.03 (19)	C19—C18—H18A	109.3
N3—C11—C12	118.1 (2)	N3—C18—H18B	109.3
C8—C11—C12	122.84 (18)	C19—C18—H18B	109.3
C19—N4—C19^i^	116.1 (3)	H18A—C18—H18B	108.0
C19—N4—H4	122.0	C13—C14—C15	119.9 (3)
C19^i^—N4—H4	122.0	C13—C14—H14	120.1
C11—C8—C7	122.52 (18)	C15—C14—H14	120.1
C11—C8—C9	130.9 (2)	C9—C10—H10A	109.5
C7—C8—C9	105.7 (2)	C9—C10—H10B	109.5
C5—C6—C1	119.9 (2)	H10A—C10—H10B	109.5
C5—C6—N1	120.5 (2)	C9—C10—H10C	109.5
C1—C6—N1	119.6 (2)	H10A—C10—H10C	109.5
C16—C17—C12	120.0 (2)	H10B—C10—H10C	109.5
C16—C17—H17	120.0	C2—C1—C6	118.8 (3)
C12—C17—H17	120.0	C2—C1—H1	120.6
N2—C9—C8	111.3 (2)	C6—C1—H1	120.6
N2—C9—C10	118.7 (2)	C3—C2—C1	121.6 (3)
C8—C9—C10	130.0 (2)	C3—C2—H2	119.2
N4—C19—C18	111.1 (2)	C1—C2—H2	119.2
N4—C19—H19A	109.4	C3—C4—C5	120.4 (3)
C18—C19—H19A	109.4	C3—C4—H4A	119.8
N4—C19—H19B	109.4	C5—C4—H4A	119.8
C18—C19—H19B	109.4	C2—C3—C4	119.1 (3)
H19A—C19—H19B	108.0	C2—C3—H3	120.4
C16—C15—C14	120.1 (2)	C4—C3—H3	120.4
			
N2—N1—C7—O1	176.4 (2)	C11—C12—C17—C16	−177.64 (19)
C6—N1—C7—O1	11.4 (3)	N1—N2—C9—C8	−1.4 (2)
N2—N1—C7—C8	−2.4 (2)	N1—N2—C9—C10	−179.79 (19)
C6—N1—C7—C8	−167.39 (18)	C11—C8—C9—N2	−168.8 (2)
C7—N1—N2—C9	2.5 (2)	C7—C8—C9—N2	0.0 (2)
C6—N1—N2—C9	169.01 (18)	C11—C8—C9—C10	9.3 (4)
C18—N3—C11—C8	−178.9 (2)	C7—C8—C9—C10	178.1 (2)
C18—N3—C11—C12	−0.7 (3)	C19^i^—N4—C19—C18	−172.3 (2)
C17—C12—C11—N3	−69.2 (3)	C17—C12—C13—C14	0.1 (4)
C13—C12—C11—N3	112.2 (2)	C11—C12—C13—C14	178.8 (2)
C17—C12—C11—C8	108.9 (2)	C14—C15—C16—C17	−0.1 (4)
C13—C12—C11—C8	−69.7 (3)	C12—C17—C16—C15	−1.0 (3)
N3—C11—C8—C7	−0.3 (3)	C1—C6—C5—C4	−1.8 (3)
C12—C11—C8—C7	−178.43 (19)	N1—C6—C5—C4	177.1 (2)
N3—C11—C8—C9	166.9 (2)	C11—N3—C18—C19	−135.6 (3)
C12—C11—C8—C9	−11.2 (3)	N4—C19—C18—N3	−52.7 (3)
O1—C7—C8—C11	−7.3 (4)	C12—C13—C14—C15	−1.3 (4)
N1—C7—C8—C11	171.45 (18)	C16—C15—C14—C13	1.3 (4)
O1—C7—C8—C9	−177.3 (2)	C5—C6—C1—C2	2.3 (3)
N1—C7—C8—C9	1.4 (2)	N1—C6—C1—C2	−176.6 (2)
C7—N1—C6—C5	−32.3 (3)	C6—C1—C2—C3	−1.3 (4)
N2—N1—C6—C5	163.56 (19)	C6—C5—C4—C3	0.3 (4)
C7—N1—C6—C1	146.5 (2)	C1—C2—C3—C4	−0.2 (5)
N2—N1—C6—C1	−17.5 (3)	C5—C4—C3—C2	0.7 (5)
C13—C12—C17—C16	1.0 (3)		

Symmetry codes: (i) −*x*, *y*, −*z*+1/2.

### Hydrogen-bond geometry (Å, °)

**Table:**

*D*—H···*A*	*D*—H	H···*A*	*D*···*A*	*D*—H···*A*
N3—H3A···O1	0.86	2.00	2.722 (2)	140
